# A Qualitative Analysis of Beekeepers’ Perceptions and Farm Management Adaptations to the Impact of Climate Change on Honey Bees

**DOI:** 10.3390/insects12030228

**Published:** 2021-03-06

**Authors:** Monica Vercelli, Silvia Novelli, Paola Ferrazzi, Giada Lentini, Chiara Ferracini

**Affiliations:** Department of Agricultural, Forest and Food Sciences (DISAFA), University of Torino, Largo Paolo Braccini 2, 10095 Grugliasco, Italy; silvia.novelli@unito.it (S.N.); paola.ferrazzi@unito.it (P.F.); giada.lentini@unito.it (G.L.); chiara.ferracini@unito.it (C.F.)

**Keywords:** honey bee, climate change, beekeeping, focus group, SWOT analysis, bee hive management, beekeeping farm

## Abstract

**Simple Summary:**

This paper addresses climate change effects on honey bees and beekeeping, as observed by the beekeepers. Focus groups were used to identify the perceptions, thoughts and impressions of two groups of beekeepers, regarding their viewpoints and direct observations on the effects of climate change on honey bees and management strategies. Beekeepers reported several consequences related to severe weather events (weakening or loss of colonies; scarcity of nectar, pollen, and honeydew; decrease or lack of honey and other bee products; intensive transhumance; greater infestation by varroa; decline in pollination), making it necessary to provide supplemental sugar feeding, more effective and sustainable techniques for varroa control, and increased production of nuclei. Thanks to their strong motivation and collaborative attitude, beekeepers succeed in adopting strategies that are able to limit the climatic adverse effects. However, the institutional and financial support for the beekeeping sector should be strengthened and better targeted in order to help beekeepers to cope with the specific issues arising due to climatic stresses.

**Abstract:**

(1) Background: Bees are the primary animal pollinators in most ecosystems, and honey bees (*Apis mellifera* L.) are important providers of pollination ecosystem services and products. Climate change is one of the major threats for honey bees. (2) Objectives and methods: Qualitative research using focus group discussions was carried out in northwestern Italy, to investigate the beekeepers’ perceptions of climate change effects, the relevant management adaptations, and the main issues affecting the sector. (3) Results: Beekeepers reported several consequences related to severe weather events (weakening or loss of colonies; scarcity of nectar, pollen, and honeydew; decrease or lack of honey and other bee products; greater infestation by varroa; decline in pollination), making it necessary to provide supplemental sugar feeding, intensive transhumance, more effective and sustainable techniques for varroa control, and increased production of nuclei. A strengths, weaknesses, opportunities, and threats (SWOT) analysis was completed, displaying the factors able to strengthen or weaken the resilience of the beekeeping sector to climate change. (4) Conclusions: Thanks to their strong motivation and collaborative attitude, beekeepers succeed in adopting farm and bee hive adaptation strategies that are able to limit the climatic adverse effects. However, these findings highlight how the institutional and financial support for the beekeeping sector should be strengthened and better targeted.

## 1. Introduction

Climate change is a global and multi-faceted phenomenon, seriously affecting the distribution and abundance for a wide range of ecosystems and organisms, including plants and pollinators. Warmer temperatures may impact on insect species’ population dynamics directly through effects on survival, life cycle, fecundity, and dispersal [1]. Climate change is reported to impact insect pollinators and their pollination activity and efficiency [2,3], with a significant decline of bees and biodiversity [4,5,6,7,8,9]. Furthermore, plant–pollinators mutualism may also be affected by global warming and consequently temporal mismatches among the mutualistic partners may occur [10,11,12,13,14]. Over 80% of wildflower species and cultivated plants strictly depend on insect pollinators, mainly bees [15,16,17], and scientists estimated the value of insect pollination ecosystem services being more than 150 billion euro/year worldwide, corresponding to about 9% of the total economic value of agricultural crops for human consumption [18,19].

Among pollinators, bees are the primary animal pollinators in most ecosystems [20], and honey bees (*Apis mellifera* L.) play a key role as providers of pollination services conserving plant biodiversity [21]. Moreover, honey bees are essential for the production of bee products. Beekeeping supplies market goods (honey, pollen, royal jelly, propolis, wax, etc.) and livestock (artificial swarms, packed bees, queens). Currently, a total of 92.3 million hives have been recorded around the world, including 17.6 million in Europe and 1.5 million in Italy [22]. Honey production amounts to around 1.8 million tons in the world, 283 thousands tons in Europe and 23 thousands tons in Italy [22].

Climate change is a severe threat to honey bees [23,24], significantly influencing other strictly related factors such as diseases, parasites, predators, parasitoids, viruses, and pesticide use [25,26,27,28]. These issues deeply influence the behavior, physiology and distribution of honey bees, and indirectly the availability of feeding sources necessary for colony survival and development [29]. In this context, beekeeping management practices are crucial for preserving the health of honey bee colonies, overwintering success, and productivity.

In recent decades, honey bees’ health has received considerable media and public attention within the European Union. As the effects of climate change strongly affect pollinators and beekeeping, this topic is deeply investigated by scientists, and specific policy measures have been implemented at the national and international levels [30,31]. To date, most of the investigations were aimed at analyzing beekeeping farms, beekeeper profit, and value chain individually [32,33,34,35], but little research concerns the climate change effects on beekeeping activity and bee hive and farm management [36,37]. Qualitative and participatory approaches, such as focus group discussions (FGDs), may contribute to help out the social interaction among stakeholders as in informal life settings [38,39,40]. FGDs were used by some researchers mainly to examine beekeeping in terms of practices, honey production and livelihood challenges for the beekeeping value chain, and to develop a social–ecological system approach [41,42,43,44,45,46].

To address this lack of knowledge, we aimed at investigating the climate change effects on honey bees and beekeeping, focusing on beekeepers and analyzing their perceptions and the management adaptation strategies adopted using a qualitative approach. The current study was performed in the frame of the Interreg V-A France–Italy (Alcotra) “CClimaTT—Climate Change within Cross-Border Territories” project, carried out in northwestern Italy. FGDs were carried out to investigate: (i) the beekeepers’ perceptions about climate change; (ii) the management strategies adopted for adverse effects mitigation; (iii) the favorable and unfavorable factors affecting the beekeeping sector.

## 2. Materials and Methods

### 2.1. Study Area

Investigations were carried out in Piedmont, a region located in NW Italy, within cross-border territories of the Interreg project. Piedmont is the most important Italian region for beekeeping, with about 4000 professional beekeepers and 2000 hobbyists, managing 175,329 and 30,258 registered hives, respectively [47], and with an estimated production of over 5000 tons of honey in 2018 [48]. We selected two areas, both located in the province of Cuneo, in the “Parco Naturale Gesso e Stura” (Area 1) and in the “Unione di Comuni Colline di Langa e del Barolo” (Area 2).

Area 1 is a protected area, which is partly flat and hilly and characterized by a mosaic of river environments, agricultural fields, and forest lands, with a prevalence of oak (*Quercus* spp.), black locust (*Robinia pseudoacacia*), chestnut (*Castanea sativa*), poplar (*Populus* spp.), alder (*Alnus* spp.), willow trees (*Salix* spp.), and ash (*Fraxinus* spp.). Beekeeping is a flourishing activity, with several professional beekeepers being owners of large farms, managing permanent apiaries, and practicing transhumance. Area 2 is an intensive wine-growing area, which is mostly hilly, with a prevalence of vineyards intermixed with hazelnut and wooded patches. Beekeeping is mainly conducted in permanent apiaries, and the transhumance involves the mobilization of a small number of bee hives. The activity is mainly carried out as a secondary activity (part-time beekeepers), and mostly by small- and medium-sized farms.

### 2.2. Survey

We set up two focus group discussions, FG1 in Area 1 and FG2 in Area 2. FGDs were conducted in November 2018; each session lasted about two hours and was audio recorded. Participants were recruited in collaboration with the partners of the CClimaTT project and selected from a network of local beekeepers involved in previous communication and participatory activities. The selection criteria included: number of bee hives owned, experience in beekeeping (years of activity), and type of beekeeping activity (professional or hobbyist). Focus groups usually consist of six to eight participants and rarely more than 12 [40,49,50]. However, some authors endorsed the use of smaller groups, i.e., three or four participants, when the group shares specialized knowledge or experience [40,51,52]. Based on the importance of beekeeping in the two areas, 11 beekeepers were recruited for FG1 and five for FG2 (Table 1).

In FG1, the participants’ age ranged between 27 and 80 years. Participants were professional, with the exception of one hobbyist, and all practicing transhumance. Nine beekeeping farms were conventional, while two were certified organic. The number of owned bee hives ranged between 24 and 1200. The group was heterogeneous also in terms of experience, including beekeepers who have been operating for more than 40 years and others who have started the activity less than five years earlier. Most of the farms were specialized in beekeeping, while two farms practiced diversified farming (e.g., cattle breeding or cultivation of small fruits). Two farms exclusively produced honey or nuclei and royal cells; the others produced both honey and nuclei, royal cells and queens. One beekeeper provided pollination services in orchards. All beekeepers sold their products to cooperatives and/or commercial companies.

Participants in FG2 were aged between 40 and 70 years. All participants were professional beekeepers. All farms were conventional and all but one practiced transhumance. Participants owned from 15 to 450 bee hives, with a diversified experience in the sector (5–50 years of activity). Three farms also cultivated hazelnut and vineyards, while two farms were specialized in beekeeping. Two farms exclusively produced honey, while the others also produced nuclei, royal cells and queens, and one provided pollination services. Besides the conventional distribution channels (wholesalers and cooperatives), two farms chose to sell directly as well.

FGDs were moderated by the researchers involved in the CClimaTT project. After following the common recommended pattern for introducing the group discussion [40], i.e., welcome, overview of the topic, research goal, and ground rules, the discussion aimed to engage the participants in three key research questions:

1. What effects of climate change have you observed on bees and beekeeping in the last 10 years?
2. Did the climate change effects lead to the adoption of mitigation strategies or change in beekeeping management practices?
3. What are the positive aspects and the main difficulties in beekeeping?

The third question was addressed to elicit the strengths, weaknesses, opportunities, and threats of beekeeping in the surveyed area, in order to complete a SWOT matrix based on the participants’ perceptions.

The first step for the analysis and interpretation of the focus group data was the transcription of the audio recordings. Then, the transcriptions were analyzed using a scissor-and-sort technique, for identifying the relevant sections to the research questions and classifying major topics and issues [53]. Responses were compared across different types of respondents and across the two areas. Finally, information was summarized and synthesized and key terms and sentences of respondents were used to support findings.

## 3. Results

### 3.1. Beekeepers’ Perceptions about the Effects of Climate Change over the Past 10 Years

Both groups of beekeepers highlighted a decrease in nectar, pollen, and honeydew resources, with a reduction or absence of some types of honey, as well as a decrease in stocks stored in the nest, compromising a successful winter survival. Nectar was reported to be often unavailable in the flowers. Unusual spring frosts negatively affected the production of the black locust, a flower species with higher market value among Piedmont honeys. In recent years, the flowering of the black locust had significantly reduced. Chestnut blossom was late and not very profitable with a reduction in honey yield, and resulting in a lighter and sweeter honey than the typical dark and bitter chestnut honey. Mountain blossom honey was also produced in small amount, due to spring frosts and then to summer drought. Some beekeepers of the group stated:

*“2017 was a disastrous year, the worst in the last 40 years for beekeeping”* (Antonio, FG1). *“I remember that twenty years ago the flowering of the black locust was a wonder, now many plants have dried up”* (Silvana, FG2). *“We forget the past with five or six supers of chestnut honey. Currently, if you get the third super it is fine”* (Antonio, FG1).

Members of FG2 remarked that in some areas the production of honeydew by *Metcalfa pruinosa* (Say) was more abundant and replaced wildflowers. With regard to fir honeydew honey, both groups argued that there was instead an increase in new areas and a decrease in areas that were previously characterized by high levels. Members of FG1 added that pollen production was also low, in particular chestnut pollen. Members of FG2 confirmed this observation and attributed the negative conditions of the colonies in spring to the consequences of the drought of the previous year. Moreover, with regard to extreme climatic events, members of FG2 added that the scarcity of water also affected the strength of the colonies and the consistency and development of the nuclei. In particular, three beekeepers claimed that:

*“Chestnut pollen production in the past amounted to about four kilos per hive and has now halved”* (Fabrizio, FG1). *“The consequences of last year’s drought affected the honey and pollen stocks in the present year”* (Silvana, FG2). *“My colonies were very weak at the beginning of the harvest period”* (Davide, FG2).

Members of FG1 noted a major harvest by honey bees of the protein-based flour distributed in the stables for livestock feeding, in spring—to mitigate the decrease of pollen sources due to spring frosts—and during summer droughts. Beekeepers reported an evident change in bee forage in favor of “new” or more abundant blooms. The nectar flow was found to be associated with the tree of heaven (*Ailanthus altissima*), the Japanese knotweed (*Reynoutria japonica*), ivy (*Hedera helix*) (an important resource before the wintering of honey bees), and the linden tree (*Tilia* spp.). Furthermore, the wild clematis (*Clematis vitalba*), previously almost neglected by honey bees, was recently reported as a foraging source, also for nectar after the chestnut blossom. Beekeepers also observed a change in the time and length of the flight activity and foraging of honey bees, specifying: *“Sometimes the honey bees only collect from 8 a.m. to 10 a.m.”* (Eraldo, FG1). 

According to FG1, the late flowering of cultivated buckwheat, *Fagopyrum esculentum*, is supposed to give nectar only when the air humidity is high; in several cases only a pollen collection was detected. Beekeepers in both FGs had different views about the occurrence of milder winters. Some of them highlighted that due to too mild winters, there was a greater infestation of the varroa mite (*Varroa destructor* Anderson and Trueman) because the queen does not naturally stop egg laying. Thus, it is difficult to effectively carry out varroa control due to the almost constant presence of the brood. On the other hand, others believed that this situation could be positive, as colonies are ready to harvest earlier. Both groups reported the discovery and spread of new predators and pathogens of the honey bees such as *Nosema ceranae* (Fries) favored by high temperatures and high humidity especially in August, with the result of a reduction in the development of colonies and a wintering of smaller colonies. Further concerns were also reported for the reproductive aspects by both groups, such as failure of the fertilization of queens, reduction of drones, and swarmings. Imbalances due to unstable climate often result in loss of colonies.

### 3.2. Mitigation Strategies and Changes in Bee Hive and Farm Management Practices

The main actions strictly related to climate change mitigation were largely shared by the beekeepers of both FGs. Beekeepers agreed that the most important action related to the lack of nectar is supplying the colonies with supplemental feeding. Syrup or candy have to be provided for their survival not only in periods with no flowering and low temperatures, such as in late Autumn and winter, but also in harvesting periods. Notably, one beekeeper from FG1 declared:

*“Last year, unlike in the past, we had to feed the honey bees in the harvesting period, otherwise they would have disappeared completely. Fortunately, beekeepers are vigilant and save their honey bees”* (Antonio, FG1). *“You can feed them as long as you want but in the end it’s just water and sugar”* (Silvana, FG2).

Members of FG1 added that there is no feasible solution to the lack of pollen due to climate change, as no adequate protein nutrition for honey bees is available. Thus, intensive transhumance became an obligatory choice, requiring mobilization of bee hives to mountainous areas in the summer. New blooms and locations can make up for the lack of resources and for the economic support of the farm. The main highlights reported by the beekeepers were:

*“Beekeepers practicing transhumance suffer less damage from climate change because the mountainous areas are cooler and richer in humidity and more productive”* (Giovanni, FG2). *“Fortunately, I have been practicing transhumance in the mountain for several years, otherwise I would have produced only black locust and maybe metcalfa honey”* (Antonino, FG2). *“In the past, after transhumance, honey bees were still able to fill one or two supers of lowland honey. On the contrary, no other honey has been collected this year”* (Antonio, FG1).

Both groups also highlighted the serious problem of parasitisation by *V. destructor*. Climate change allows an extension of queen oviposition in winter, causing a continuous reproduction of the mite. Thus, the effectiveness of control is compromised, and further actions are required (e.g., artificially interrupting brood production through seasonal and winter queen caging, and total brood removal). Members of FG2 also remarked that the control of varroa requires the use of various products such as synthetic acaricides and oxalic and formic acids, and the postponement of the treatment period due to the climate: *“When the winters are mild, the varroa infestation level is very high, and the mite becomes more difficult to control”* (Sergio, FG2). *“High temperatures in winter do not allow honey bees to stop their activity”* (Sergio, FG2). *“We decided to use formic acid at the end of July, but we had to postpone the treatment by one month due to the high temperatures during the night, with the risk of compromising the varroa control efficacy”* (Giovanni, FG2). Both FGs highlighted the need to frequently replace queens, often due to unsuccessful fertilization. In order to maintain the bee stock and consequently the farm viability, beekeepers had to increase the production of nuclei. Moreover, they had to leave the latest honey stock available, for having stronger colonies, and make the equalization of colonies, even in periods other than autumn. One beekeeper from FG2 concluded: *“Last year the situation was dramatic, I don’t know what more we could have done*” (Sergio, FG2).

### 3.3. A SWOT Analysis of the Beekeeping Sector in the Surveyed Area

A discussion was stimulated in order to draw out the favorable and unfavorable factors affecting the beekeeping sector, which are able to strengthen or weaken its resilience to many threats, in particular climate change.

Results were organized in the form of a SWOT matrix (Table 2), identifying strengths and weaknesses (i.e., internal factors that the operators of the sector have some control over and can try to change or manage) and opportunities and threats (i.e., external factors, deriving from the environmental, market, or regulatory context and thus not depending on the organizational decisions of the beekeepers [54]. 

## 4. Discussion

Honey bees provide an essential pollination ecosystem service for the conservation of plant biodiversity and the provision of agricultural goods [18,19,55]. Due to these reasons, it is necessary to identify all threats afflicting honey bees and beekeeping and to find suitable solutions, especially with regard to the impact of the climate change that occurred in recent decades.

Beekeeping has important economic impacts, not only for the market of bee products but especially for the pollination of a huge number of cultivated and spontaneous plants, thanks to the conspicuous honey bee stock present worldwide [22]. Nevertheless, over the last years several authors have observed and estimated an alarming decline in pollinators (honey bees included) and in the value of the relevant market products, identifying climate change as one of the main current threats [5,16,24]. In particular, market reports show a decrease in honey and other bee products [30]. Despite this, the demand for honey worldwide [22,56] and in Europe is growing, and consumers are showing a growing interest in high-quality and organic honey. However, climate change affects both the biology and behavior of honey bees and their production, impacting bee hive and farm management as well [9,57]. Previous studies analyzing the adoption of different management practices and their determinants were few and usually relied on structured or unstructured interviews [36,58,59]. 

In this paper, two FGDs were used to identify the perceptions, thoughts, and impressions of selected groups of beekeepers, involving them in a conversation aimed at exchanging viewpoints and direct observations about the effects of climate change on their activity. The analysis benefited from the realistic responses of the participants, the opportunity to compare opinions among reference group members and the two FGs, and the feedback effect among the group that spurred further reflections on the suggested topics. Through the FGs, the beekeepers discussed the effects of climate change they observed on honey bees and beekeeping activity. Moreover, the participants highlighted how they had to modify their management practices, adopting specific actions to mitigate the adverse effects of climate change. Albeit operating in territories characterized by different land use and economic opportunities (a natural park and an intensive wine-growing area), beekeepers from both areas reported several common climate change effects and threats affecting their activities. In their opinion, severe weather events (e.g., high temperatures in winter and summer, spring frosts, decreased rainfall, drought, and heavy water floods) show their effects in terms of direct and indirect damages. Direct damages include the weakening or loss of honey bee colonies, greater infestation by *V. destructor*, and new biotic threats. As indirect damage, beekeepers reported the widespread scarcity of nectar, pollen and honeydew resources, the considerable reduction (or lack) of honey and other bee products production, and the decline in pollination activity.

To cope with these issues, beekeepers have to adopt many new practices and measures for maintaining the honey bee colony strength and the farm income, such as supplemental sugar feeding to make up for the lack of nectar and therefore save the bees; the adoption of more effective as well as sustainable techniques for varroa control (e.g., queen caging and total brood removal) [60,61]; intensive transhumance practice; and increased production of nuclei [35]. The adoption of the adaptation strategies entails higher costs, both variable and fixed, in particular out-of-pocket expenses for supplemental feeding and intensive transhumance, and additional labor costs, since the adaptation practices are time-consuming and labor-intensive.

The main issues that emerged in the brainstorming session were used to fill in a SWOT matrix, identifying internal and external factors affecting the capacity of the sector to find adaptation strategies. The main strength of the beekeeping activity was found to be in the personal traits of beekeepers, such as their strong motivation and satisfaction in their work. Their passion and commitment, together with the collaborative spirit between and within generations, was reported as one of the main drivers for resilience in the sector. These internal factors are boosted by the growing public interest for honey bees (as “environmental sentinels”) and for high-quality unifloral and multifloral honeys. Unfavorable factors are related to climatic, anthropic and biotic stresses (e.g., the low average production and the reduced strength of the colonies) as well as to institutional or market issues (e.g., the lack of adequate institutional and financial support and the competition with low-quality and cheap bee products). Furthermore, the beekeepers acknowledged well-known structural weaknesses of the sector. Organizational issues, a lack of financial resources and technical endowments, a shortage of both family labor and skilled seasonal labor, poor marketing strategies, and lack of sales skills, weaken the ability to withstand climatic and market adversities.

The SWOT analysis is the basis to generate discussion about the strategies and the policies able to address the current challenges faced by the beekeeping sector. With special reference to climate change effects, a significant support to the sector may derive from a more effective public intervention. Beekeepers strongly complain of the lack of specific policy measures and funding in relation to climate change. According to them, the European policies (e.g., Reg. (EU) No. 1308/2013 and Reg. (EU) No. 2015/1368) mainly provide support for the purchase of hive boxes, equipment, and machinery. The beekeeping sector, instead, would rather that financial support be focused on additional operating costs relating to the implementation of specific adaptation strategies (e.g., out-of-pocket expenses for supplemental feeding and the transhumance practice). The discontent of the beekeepers is explained in such a sentence: *“The brown marmorated stink bug was reported and the public support arrived immediately, since more profitable agricultural sectors receive much more attention. Varroa in honey bees is a problem we have to face alone”* (Sergio, FG2).

Apart from the aspects strictly related to climate change, other issues dealing with bee hive and farm management and honey production have also emerged. These include intensive agriculture including continuous cropping with crops not useful for pollinating insects [62,63,64,65]; the reduction of pasture meadows with the consequent loss of blooms widely present in the past such as dandelion (*Taraxacum officinale*) or white clover (*Trifolium repens*) all providing forage for typical honey; the use of chemicals (e.g., neonicotinoids) and herbicides (e.g., glyphosate) [66,67,68,69,70,71,72,73,74] applied in pre-flowering or flowering without respecting the imposed restrictions; the cultivation of hybrids, such as sunflower (*Helianthus annuus*) and buckwheat, important resources during the summer, which do not provide nectar; the release of the parasitoid *Neodryinus typhlocybae* (Ashmead), controlling *M. pruinosa* populations [75], and consequently the production of honeydew and honeydew honey; and the loss of plant biodiversity. On the other hand, the abandonment of the woodlands may allow the spread of useful plants such as cherry (*Prunus avium*) and ivy, offering an important nectar flow for honey bees, as one beekeeper observed: *“Recently, an important source of nectar is the cherry tree, which was not very common until ten years ago. Cherry honey is now the main spring production and replaced dandelion”* (Carlo, FG1).

The variations occurring in the flora visited by bees over the last few decades [76] were reported by the beekeepers of both FGs and also confirmed in the melissopalynological, physical-chemical and sensory analyses of current honeys compared to those produced in the Area 1 analyzed in the past (P.F., personal investigations).

## 5. Conclusions

The present study used a qualitative approach based on beekeepers’ perceptions for highlighting the threats induced by climate change on honey bees and bee hive and farm management in two different areas characterized by different agricultural intensity in northwestern Italy. Moreover, mitigation strategies and changes in the management practices have been addressed. The analysis was performed in accordance with the guidelines of national and EU initiatives aimed at promoting the conservation of biodiversity and the protection of pollinators (e.g., honey bees, wild bees, butterflies) through different targeted actions, also involving the participation of beekeepers and farmers [77,78]. Currently, beekeepers operating in the two investigated areas succeed in adopting strategies that are capable of limiting the climatic adverse effects, thanks to their strong motivation and collaborative attitude. However, in a context where running and labor costs for implementing the adaptation strategies are bound to rise, the institutional and financial support for the sector should be strengthened and better targeted in order to help beekeepers cope with the specific issues arising due to climatic stresses. 

## Figures and Tables

**Table 1:** Main farm characteristics and management practices adopted by the participants of the two focus group discussions (FGDs) (n).

Group	Participants	Bee Hives	Transhumance	Organic	Diversified Farming	Direct Sales
FG1	11	24–1200	All	2	2	none
FG2	5	15–450	4	none	3	2

**Table 2:** Strengths, weaknesses, opportunities, and threats (SWOT) matrix of the beekeeping sector in the surveyed area.

Strengths	Weaknesses
- Strong motivation of beekeepers - Collaboration among beekeepers - Collaboration among different generations of beekeepers - Advances in genetic selection	- Lack of time and labour for facing the adoption of new time-consuming and labour-intensive practices (e.g., new practices to control the varroa mite) - Lack of financial resources for bearing higher management costs (e.g., supplemental feeding and intensive transhumance) - Scarce technical endowments (i.e., equipment and machineries) - Lack of expertise among the younger generations of beekeepers - Lack of effective marketing strategies in the beekeeping sector - Lack of beekeeper skills for the sale of hive products
**Opportunities**	**Threats**
- Recent increase of retail prices of honey bee products (FGs took place in 2018, a year characterized by an exceptional increase in the selling prices) - Public interest in bees as environmental bioindicators (“environmental sentinels”) - Consumers’ demand for typical honey productions (e.g., mountain blossom honey)	- Reduced strength of the honey bee colonies due to climatic, anthropic (pesticides), and biotic stresses - Significant decline in the production of honey and other bee products (e.g., pollen) - Increase of varroa infestation and other diseases (e.g., *Nosema ceranae*) - Failure in honey bee queen mating - Insufficient and mistargeted public financial support to the beekeeping sector - Lower prices of low-quality honey supplied by foreign competitors on the market - Lack of knowledge transfer and technical advisory services for the beekeeping sector at the regional and national levels
